# Robust Nanofiber Mats Exfoliated From Tussah Silk for Potential Biomedical Applications

**DOI:** 10.3389/fbioe.2021.746016

**Published:** 2021-12-02

**Authors:** Ming Chen, Jianzhong Qin, Shijun Lu, Feng Zhang, Baoqi Zuo

**Affiliations:** ^1^ The Affiliated Stomatological Hospital of Soochow University, Suzhou Stomatological Hospital, Suzhou, China; ^2^ College of Textile and Clothing Engineering, Soochow University, National Engineering Laboratory for Modern Silk, Suzhou, China; ^3^ Department of Orthopedics, The Second Affiliated Hospital of Soochow University, State Key Laboratory of Radiation Medicine and Protection, Soochow University, Suzhou, China; ^4^ State Key Laboratory of Biotherapy, West China Hospital, West China Medicine School, Sichuan University, Chengdu, China

**Keywords:** tussah silk, nanofiber, nonwoven mats, mechanical properties, biocompatibility

## Abstract

Nanofibers as elements for bioscaffolds are pushing the development of tissue engineering. In this study, tussah silk was mechanically disintegrated into nanofibers dispersed in aqueous solution which was cast to generate tussah silk fibroin (TSF) nanofiber mats. The effect of treatment time on the morphology, structure, and mechanical properties of nanofiber mats was examined. SEM indicated decreasing diameter of the nanofiber with shearing time, and the diameter of the nanofiber was 139.7 nm after 30 min treatment. These nanofiber mats exhibited excellent mechanical properties; the breaking strength increased from 26.31 to 72.68 MPa with the decrease of fiber diameter from 196.5 to 139.7 nm. The particulate debris was observed on protease XIV degraded nanofiber mats, and the weight loss was greater than 10% after 30 days *in vitro* degradation. The cell compatibility experiment confirmed adhesion and spreading of NIH-3T3 cells and enhanced cell proliferation on TSF nanofiber mats compared to that on *Bombyx mori* silk nanofiber mats. In conclusion, results indicate that TSF nanofiber mats prepared in this study are mechanically robust, slow biodegradable, and biocompatible materials, and have promising application in regenerative medicine.

## Introduction

Silk is a kind of natural polymer secreted by silkworms. It is mainly divided into mulberry silk and wild silk. Silk fibroin has good biocompatibility, blood compatibility, biodegradability, and excellent mechanical properties (Wang et al., 2008; Kundu et al., 2013). So, it is regarded as one of the best choices of bioengineering materials and is widely developed for use in artificial skin, blood vessels, nerves, bones, ligaments, tendons, and corneas (Holland et al., 2019; Sun et al., 2021). Compared with *Bombyx mori* silk fibroin (BSF), tussah silk fibroin (TSF) contains many polar amino acids with positive charge and contains the Arg-Gly-Asp (RGD) tripeptide sequence which is a signal of cell adhesion recognition (He et al., 2013; Silva et al., 2019).

Silk fibroin has good forming and processing performance and has been processed into biological scaffolds with various shapes, such as nanofibers, porous sponges, hydrogels, microspheres, and films (Lammel et al., 2010; Rockwood et al., 2011; Gorenkova et al., 2019). Among them, silk fibroin nanofibers have been increasing attention because of its simulation of extracellular matrix (ECM) (Babitha et al., 2017; Chouhan et al., 2017; Chen et al., 2018). Currently, some processes, such as electrospinning, self-assembly, chemical dissolution, and physical fiber separation, have been developed to prepare silk fibroin nanofibers (Yin et al., 2017; Yang et al., 2018a; Humenik et al., 2018; Niu et al., 2018; Tan et al., 2018; Zheng et al., 2018; Lv et al., 2019; Cai et al., 2020). Sukigara et al. (Sukigara et al., 2003) prepared silk fibroin nanofibers with an average diameter of less than 100 nm using electrospinning. Lu et al. (Lu et al., 2011) prepared silk fibroin nanofibers with a diameter of about 20 nm by the self-assembly technique to construct silk fibroin nanofiber–based films, porous scaffolds, and injectable gels. In our previous study, calcium chloride/formic acid compound solvent has been used to dissolve silk into nanofibril directly (Zhang et al., 2014; Yang et al., 2018b). Zhao et al. (Zhao et al., 2007) employed the ultrasonic technique to directly extract nanofibers with a diameter of 20–60 nm from natural materials such as spider silk, silkworm silk, collagen, chitin, cotton, bamboo, wood, and ramee and hemp nanofibers. Silk scaffolds composed of nanofibers display superior bioactivity and thus are a more promising biomaterial for tissue engineering application. These nanofibrous materials can be used not only as scaffolds for tissue repair directly but also to study the interaction mechanism between ECM and cells *in vitro*, thus providing theoretical guidance for the design and construction of bioactive scaffolds (Kim et al., 2005; Bai et al., 2014).

However, the aforementioned methods are mainly applicable to *B. mori* silk, and the preparation methods of TSF nanofibers are still limited. Electrospinning (Liu et al., 2020) and chemical–physical combination (Zheng et al., 2018) are the two reported methods to prepare TSF nanofibers. In our previous study, the TSF nanofiber with a diameter of 611 nm was prepared by electrospinning, and the mechanical properties of the nanofiber mats were improved by chemical cross-linking (Liu et al., 2012). Zheng et al. (Zheng et al., 2018) first used sodium hypochlorite aqueous solution to separate silk fibers into millimeter-sized fragments to break the interfacial forces between the fibers and then used mild ultrasonic treatment to obtain silk fibers of different sizes. The resulting nanofibers retained the original mechanical properties of natural silk, such as the modulus. However, the preparation methods have some shortcomings, such as complex process, toxic solvent, expensive solvent, coarse fiber diameter, and poor mechanical properties (Mehrotra et al., 2019). These problems limit the development and application of TSF nanofibers. TSF–based materials, especially TSF nanofibers, can accelerate cell adhesion and proliferation *in vitro* and promote tissue regeneration *in vivo*, thus having promising applications in biomedical areas (Sahu et al., 2015; Li et al., 2017; Liu et al., 2020). Given the potential application, it is necessary to develop a new method to prepare TSF nanofibers, and the method should be simple, green, mild, and environment friendly.

In this study, we directly used the high-speed physical shearing process to destroy the connection between the tussah silk and gradually disintegrated it into nanofibers. These TSF nanofibers can be uniformly dispersed in the aqueous solution for more than one month. The nanofiber mats can be easily prepared from the TSF nanofiber solution by the simple film drying process. The morphology, molecular structure, mechanical properties, and degradation properties of the TSF nanofiber mats were characterized and analyzed by scanning electron microscopy, infrared spectroscopy, X-ray diffraction, mechanical stretching, and enzyme degradation. In addition, we evaluated the cellular biocompatibility of TSF nanofiber mats by cell culture and compared it with BSF nanofiber mats.

## Materials and Methods

### Materials

Tussah and *B. mori* silk (Haian County So HO SILK-MAKING Co., Ltd.), anhydrous sodium carbonate (Na_2_CO_3_, China National Pharmaceutical Group Corporation), protease XIV (active unit: 3.5 U/mg, Sigma-Aldrich), glutaraldehyde (50%, Sinopharmaceutical Group), anhydrous ethanol (Sinopharmaceutical Group), mouse embryonic fibroblasts (NIH-3T3, Beina Chuanglian Biotechnology Co., Ltd.), DMEM high-sugar medium (Gibco), fetal bovine serum (Procell), amino benzylpenicillin (Sigma), 0.25% trypsin digestion solution (Sigma), dimethyl sulfoxide (DMSO, Sigma), paraformaldehyde (Sigma), and CCK-8 kit (Shanghai Biyuntian Biotechnology Co., Ltd.) were used in this study.

### Fabrication of TSF Nanofiber Mats

**FIGURE 1:**
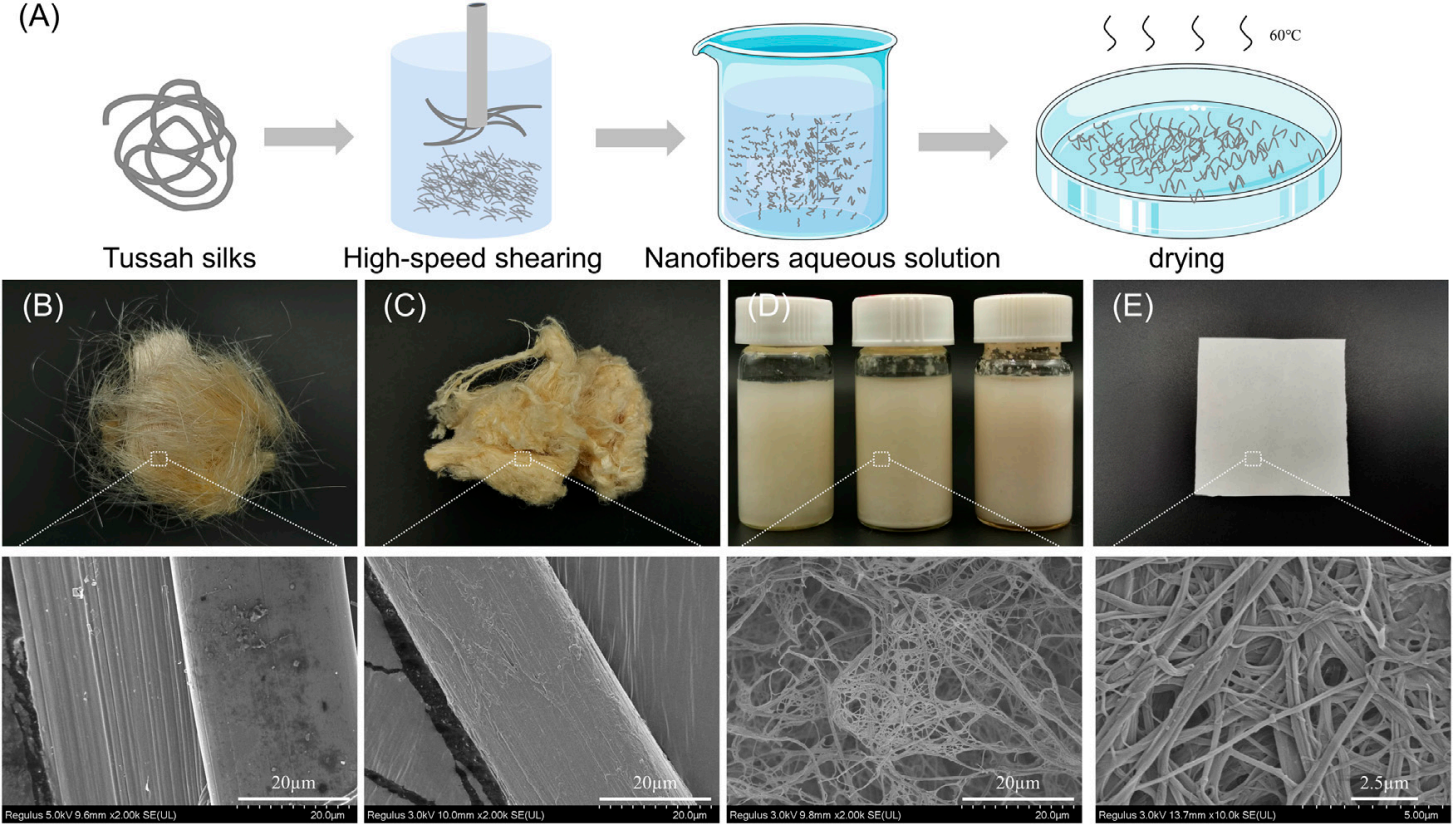
**(A)** Preparation process of the TSF nanofiber mat **(B)** Optical and SEM photos of original tussah silk **(C)** Optical and SEM photos of degummed tussah silk **(D)** Optical and SEM photos of aqueous solution of TSF nanofibers **(E)** Optical and SEM images of the TSF nanofiber mat.

As shown in Figure 1A, the tussah silk was degummed three times in boiling 0.5 wt% Na_2_CO_3_ solution for 30 min with a bath ratio of 1:50 (w/v) and rinsed thoroughly with deionized water to remove sericin. The degummed silk was cut into short fibers with about 5 mm length. These fibers were physically sheared in a high-speed shearing machine (Joyoung, Shandong, China) at 32,000 r/min for 10 min (10TSF), 20 min (20TSF), and 30 min (30TSF) to obtain TSF nanofiber solution. The solution was poured onto the Petri dish (90 mm diameter). After drying, the TSF nanofiber mats were prepared. The *B. mori* silk fibroin (30BSF) nanofiber mats were also prepared using the same process as a control in the biocompatibility experiment.

### *In vitro* Enzymatic Degradation

The 30 TSF nanofiber mats (n = 3 per group and time point) were incubated at 37°C in 0.1 U/mL protease XIV in phosphoric acid buffer solution at a ratio of 1:25. The solution was changed every 3 days, and the degraded samples were collected at designated time points. After drying, the samples were observed by SEM, and the mass remaining (MR) after degradation was calculated using the following equation:

$$\text{MR}\left(\text{\%}\right)=\frac{{\text{W}}_{\text{b}}}{{\text{W}}_{\text{a}}}\times 100\text{\%}\text{.}$$

In the formula, MR represents mass remaining, W_b_ represents the sample weight before degradation, and W_a_ represents the sample weight after degradation.

### Characterization of TSF Nanofiber Mats

The morphology of TSF nanofiber mats was observed by scanning electron microscopy (SEM) (8,100, Hitachi, Tokyo, Japan) after sputter-coated with gold for 90s. The Fourier transform infrared (FTIR) spectra of TSF nanofiber mats were obtained using an ATR-FTIR spectrometer (NicoleT iS5, Thermal Nicolet Company, United States) in the spectral region of 400–4,000 cm^−1^. The XRD spectra of TSF nanofiber mats were performed with an X-ray diffractive analyzer (40 kV, 40 mA) (Panalytical, Netherlands). The tensile properties of TSF nanofiber mats (50 mm × 10 mm) were measured using an Instron5967 universal material testing machine (Boston, United States) at 25 ± 0.5°C and 60 ± 5% relative humidity. The experiment was conducted at a cross-head speed of 10 mm/min with a gauge length of 30 mm and pretension of 0.2cN. At least five measurements for each sample were performed. The JY-PHA type contact angle tester was used to test water contact angle. The TSF nanofiber mats were randomly selected and cut into 5 cm × 5 cm, and then pasted on the contact angle tester platform. The high-precision CCD camera shot the image and sent it to the computer to display the high-definition image, and the computer automatically processed the relevant data.

### Biocompatibility

The 30TSF nanofiber mats and 30BSF nanofiber mats with a diameter of 6 mm were prepared and sterilized at high temperature and high pressure. The mouse embryonic fibroblasts (NIH-3T3) were seeded at a density of 5×10^4^ cells (10 μl) on the mats. The cell was grown on the culture plate as a control. The cell culture medium was changed every 48 h. At the designated time points (1, 3, and 7 days; *n* = 3 for each time point), the culture medium was sucked out, and 250 μl cell counting kit-8 (CCK-8) solution was added to each well and incubated in a cell incubator for 2 h. After that, 100 ml supernatant was removed from each well and added to a 96-well plate and transferred to a microplate to detect the absorbance value (OD) at 450 nm. The cell morphology on nanofiber mats was observed by SEM at 3 day and 7 day.

### Statistical Analysis

One-way analysis of variance (ANOVA) was used for comparison between groups. The *t*-test was used for comparison between the two groups. The data obtained are expressed as mean ± standard deviation. SPSS20.0 software (IBM SPSS Statistics, US) was used to analyze statistically significant difference defined as *p* < 0.05 (*), *p* < 0.01 (**), and *p* < 0.001 (***).

## Results and Discussion

The nanofibril is an important part of the hierarchical structure of native silk and contributes significance to the outstanding mechanical properties of silk (Giesa et al., 2011). Recently, the nanofibril has been successfully extracted to construct silk-based material with the nanofibrous structure for applications in biomedicine, electronic, filtration, heat retention, etc. Compared to *B. mori* silk, tussah silk showed enhanced cell growth and tissue repair likely due to its natural Arg-Gly-Asp (RGD) motifs (Sahu et al., 2015). In this study, tussah silk was selected to extract natural nanofibers to form nonwoven mats for potential biomedical application.

### Morphology of TSF Nanofibers

Figure 1 shows the process to isolate tussah silk fibroin (TSF) nanofibers. First, Na_2_CO_3_ was used to extract TSF while removing sericin and impurities. Then, the degummed tussah silk was split into nanofibers by physical shearing. The degummed tussah silk is about 30 μm, presenting a ribbon-like shape with nanofibrils stacked neatly and tightly (Shimanovich et al., 2018). The micro-sized tussah silk consists of a bundle of nanofibrils with the diameter of ∼15 nm. These nanofibrils can be separated by the physical or chemical method due to their weak adhesion (Zhang et al., 2018). To isolate these TSF nanofibers, the degummed silk was treated by physical shearing to break the weak interfaces between nanofibrils.

**FIGURE 2:**
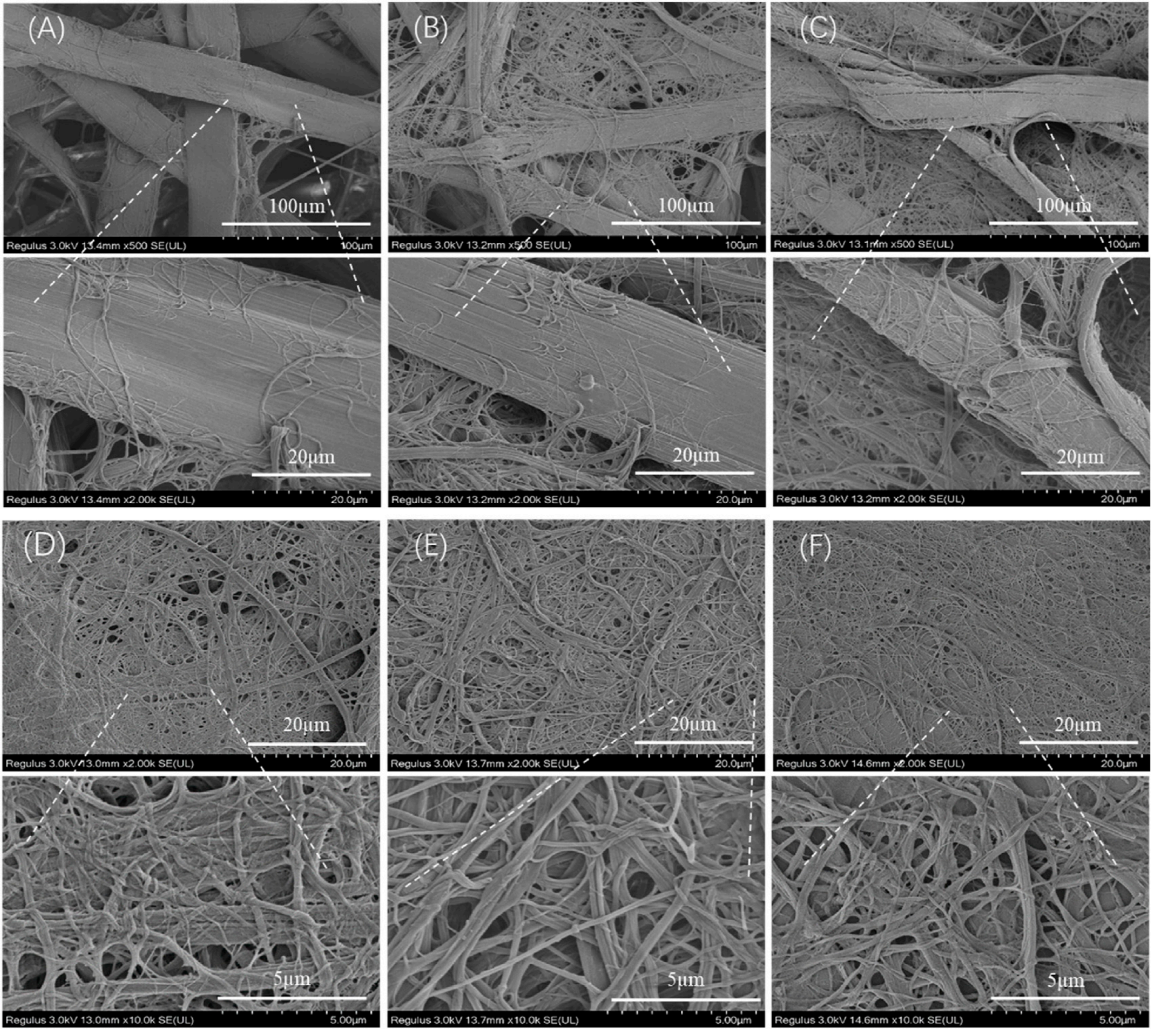
SEM observation of the downsizing process of tussah silk with shear times: 2 min **(A)**, 4 min **(B)**, 8 min **(C)**, 10 min **(D)**, 20 min **(E)**, and 30 min **(F)**.

**FIGURE 3:**
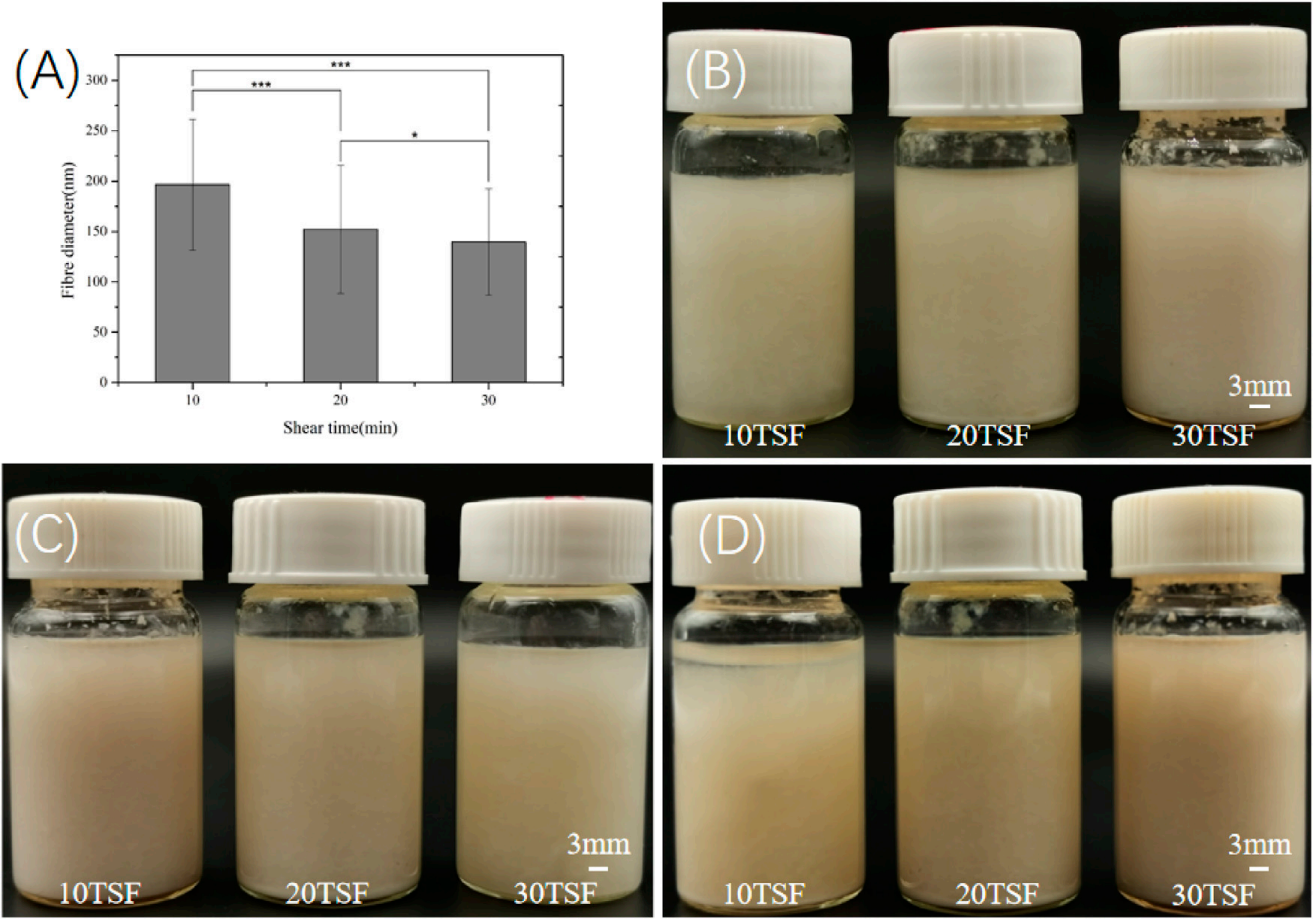
**(A)** Diameter of the TSF nanofiber after 10-, 20-, and 30-min physical shearing (*p < 0.05, **p < 0.01, ***p < 0.001) **(B)** Optical photos of the TSF nanofiber solution stored for 0 (B), 15 **(C),** and 30 **(D)** days.

Figure 2 shows the morphological change of degummed tussah silk with physical shearing. The increased microfiber and nanofiber were isolated from tussah silk with the increase of physical shearing time. Obviously, the diameter of isolated nanofibers was closely related to the shearing time. The fiber split and nanofiber exfoliation occurred after 2–8 min physical shearing (Figures 2A–C). With the increase of shearing time to 10 min, the intact tussah silk disappeared. The diameter of the isolated nanofiber decreased from 196.5 ± 65.1 nm to 152.2 ± 63.7 nm and 139.7 ± 52.9 nm with the increase of physical shearing time from 10 to 20 min and 30 min, respectively (Figure 3A). Of note, the diameter decrease is significant when the physical shearing time is increased from 10 to 20 min, but it is hard to further reduce the fiber diameter significantly through prolonging treatment time. The resulting TSF nanofibers can be stably and uniformly dispersed in aqueous solution for at least one month due to the electrostatic repulsion between anionically charged TSF nanofibers (Figures 3B–D) (Zheng et al., 2018). The TSF nanofiber solution after 10-, 20-, and 30-min physical shearing was cast to form TSF nanofiber mats for further study, including secondary structure, mechanical property, *in vitro* degradation, and biocompatibility.

### Structure of TSF Nanofiber Mats

**FIGURE 4:**
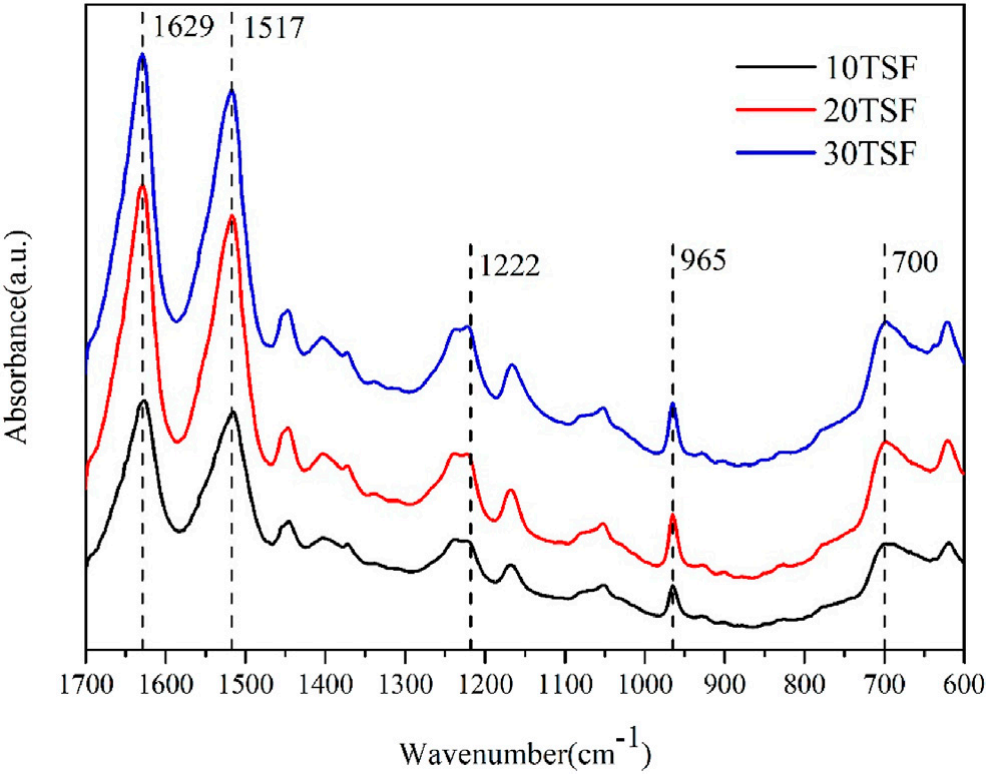
FTIR spectra of TSF nanofiber mats after 10-, 20-, and 30-min physical shearing.

**FIGURE 5:**
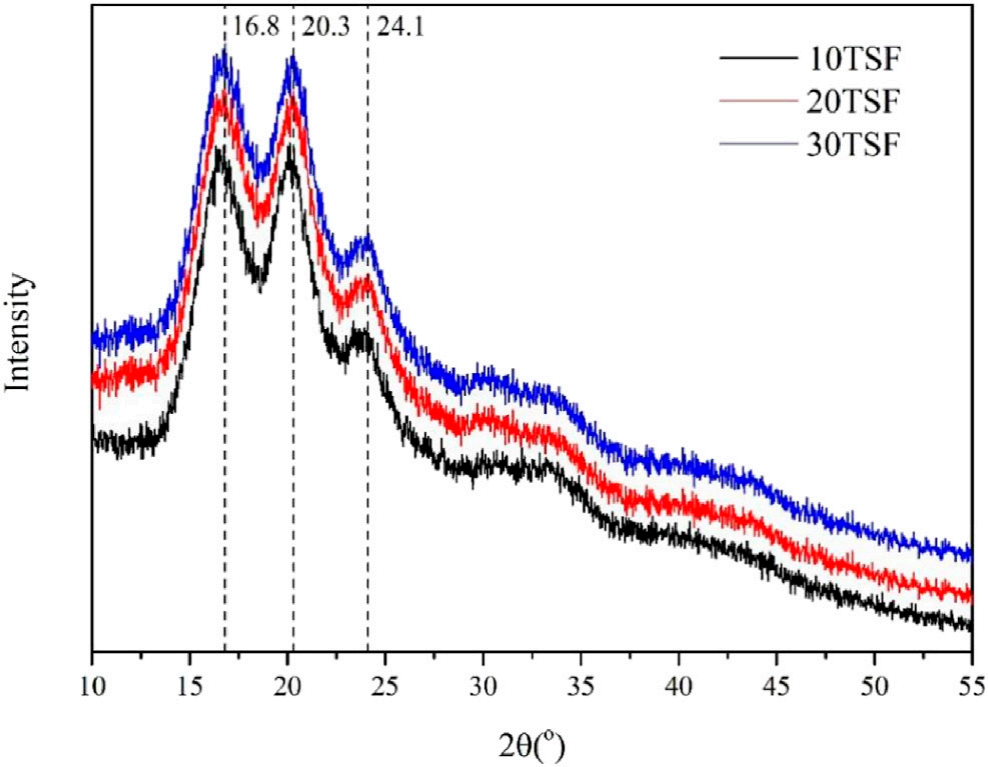
XRD pattern of TSF nanofiber mats after 10-, 20-, and 30-min physical shearing.

The FTIR spectroscopic analysis was carried out for TSF nanofiber mats to determine their secondary structure, as shown in Figure 4. All the TSF nanofiber mats showed prominent amide I, II, III, IV, and V peaks at 1629 cm^−1^, 1,517 cm^−1^, 1,222 cm^−1^, 965 cm^−1^, and 700 cm^−1^, respectively, attributed to the β-sheet conformation (Yang et al., 2018b; Fang et al., 2017), which revealed that the physical shearing had no effect on their secondary structure. To further determine the secondary structure of TSF nanofiber mats, the XRD analysis was also conducted, as shown in Figure 5. The XRD patterns of TSF nanofiber mats showed diffraction peaks around 16.8°(0.53 nm), 20.3°(0.44 nm), and 24.1°(0.37 nm) assigned to β-sheet structure, confirming the similar result to the FTIR analysis (Fu et al., 2011). It is consistent with previous studies that the physical shearing breaks only the weak interfaces between nanofibers in silk but does not destroy the strong β-sheet crystal structure in nanofibers (Okahisa et al., 2019; Wang et al., 2020a; Narita et al., 2020). So, the secondary structure of silk fibroin can be well retained by this moderate approach.

### Mechanical Properties of TSF Nanofiber Mats

**FIGURE 6:**
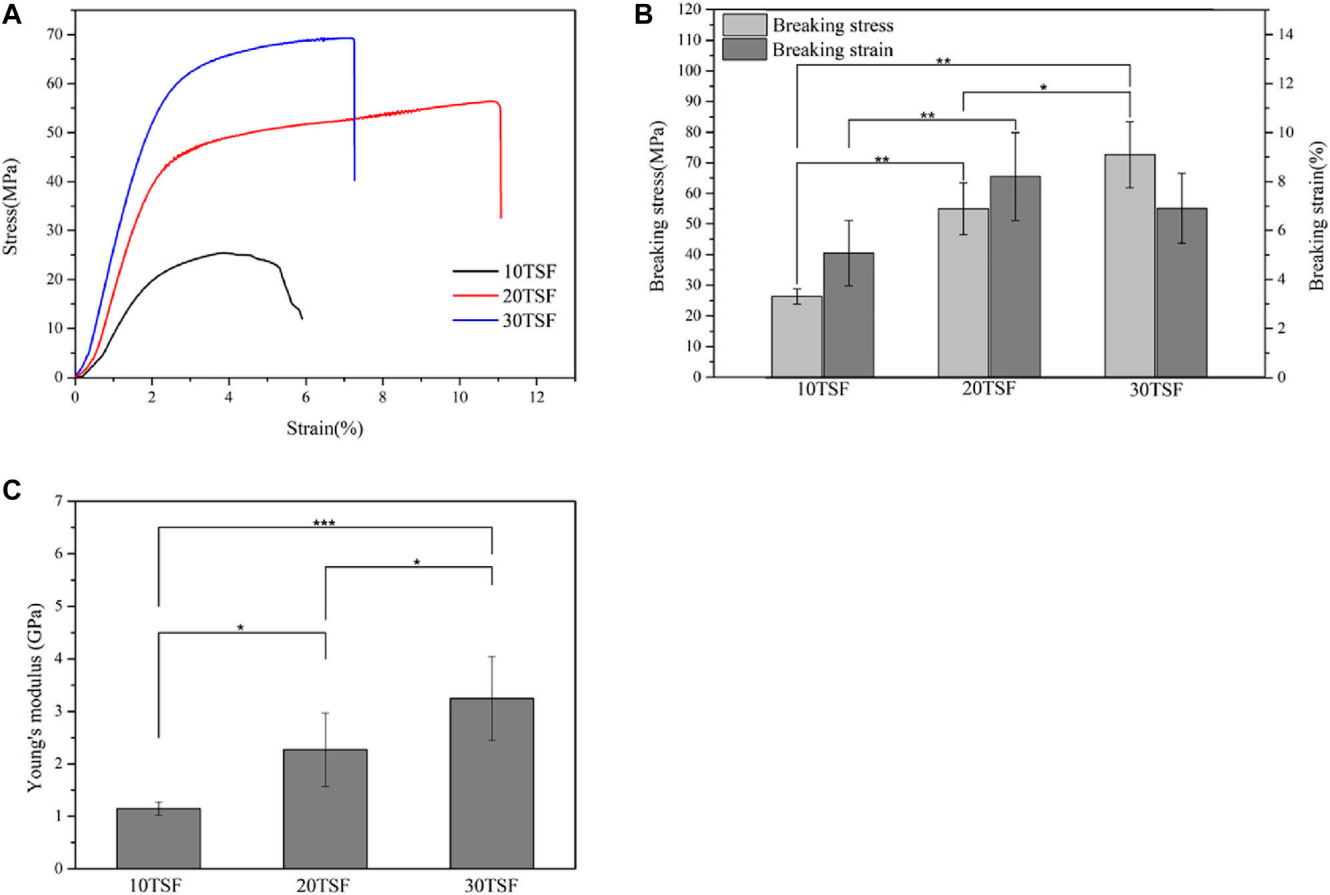
Mechanical properties of TSF nanofiber mats after 10-, 20-, and 30-min physical shearing **(A)** Stress–strain curves **(B)** Breaking stress and breaking strain **(C)** Young’s modulus of the nanofiber mats (*p < 0.05, **p < 0.01, ***p < 0.001).

**FIGURE 7:**
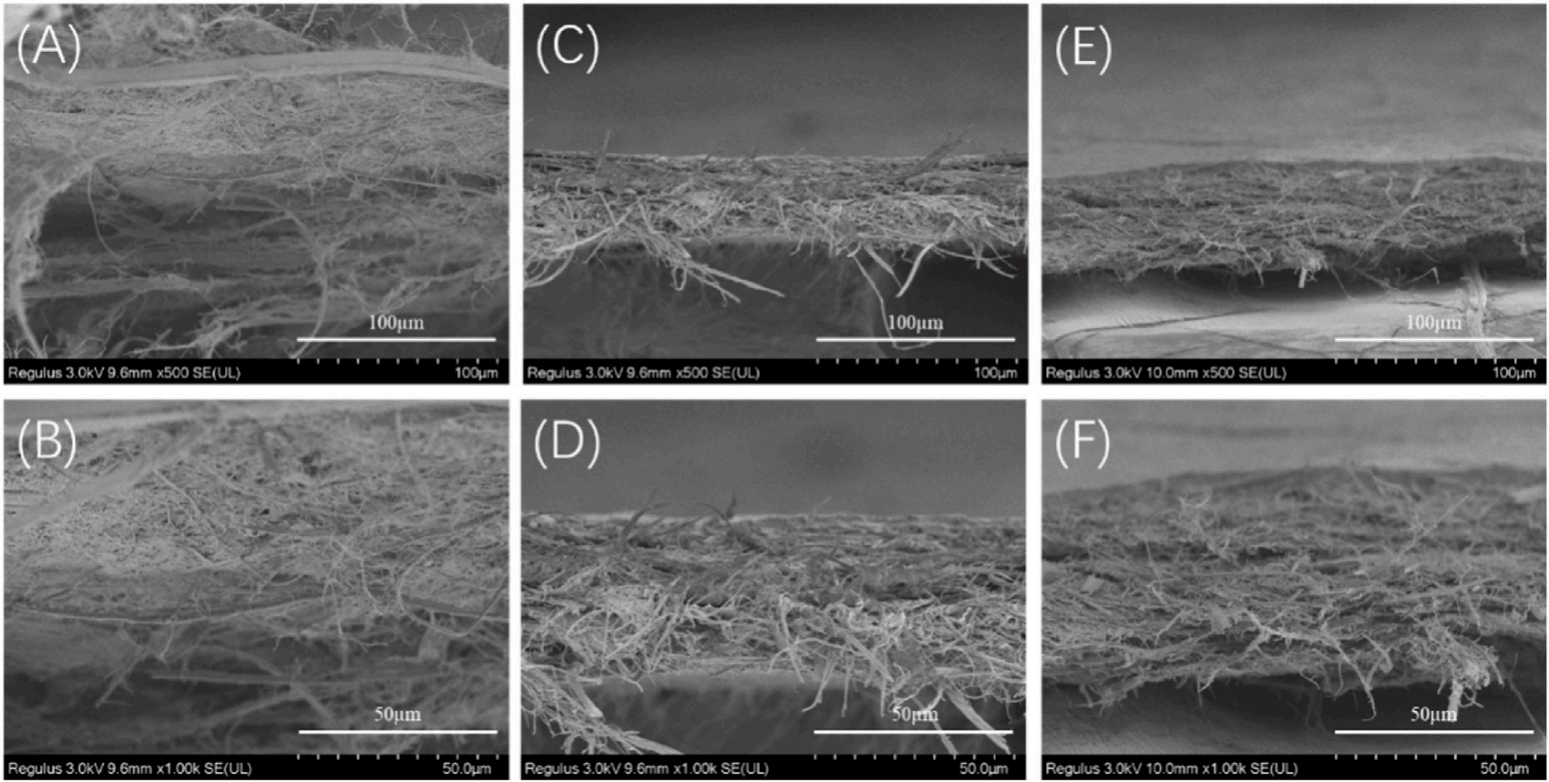
SEM images of the cross section of the TSF nanofiber mats after mechanical tensile **(A, B)** 10TSF nanofiber mat **(C, D)** 20TSF nanofiber mat **(E, F)** 30TSF nanofiber mat.

The mechanical properties of the biomaterial are important for its application in biomedicine. Figure 6 shows the stress–strain curve, breaking stress and breaking strain, and modulus of TSF nanofiber mats. The stress–strain curves of TSF nanofiber mats had a yield point followed by mild strain hardening, which was reminiscent of B. mori silk (Fu et al., 2011). The breaking stress was found to be 26.31 ± 2.54 MPa, 54.98 ± 3.79 MPa, and 72.68 ± 10.77 MPa for TSF nanofiber mats with 10-, 20-, and 30-min physical shearing, respectively. These values were better than the previous results from electrospinning BSF mats and electrospinning TSF nanofiber mats (Li et al., 2017; Shao et al., 2016; Wang et al., 2017). For example, the random and aligned electrospun TSF nanofibers showed much lower breaking stress of 1.7 and 2.7 MPa (Shao et al., 2016). The breaking stress in the range of approximately 72 MPa represented the strongest silk nanofiber mats to date, possibly due to the preservation of hierarchical structure and the dense packing of TSF nanofibers as observed by SEM (Figures 2, 7). Although the breaking strain (the highest value is 8.2%) was low, the TSF nanofiber mats were flexible which was likely due to the nanofiber nature and nanofibrous structure (Wang et al., 2020b). Young’s modulus of TSF nanofiber mats increased with the increase of shearing time, and the highest value exceeded 3 GPa, which was close to that of silk film (Zhang et al., 2015).

To further understand the failure behavior of TSF nanofiber mats, we conducted SEM imaging of the cross sections after tensile fracture, as shown in Figure 7. The fracture surfaces of TSF nanofiber mats were very rough due to their nonwoven nanofiber structure. The interface was not flat, and the individual nanofiber protrusion could be identified, likely originating from the weak bound interfaces of nanofibers. The morphological feature of cross sections clearly confirms the fracture mechanism of nanofiber fracture and nanofiber pull-out. The nanofiber fracture contributes to the high breaking stress of TSF nanofiber mats, and the nanofiber pull-out responds for its flexibility. The TSF mats derived from 10-min physical shearing was composed of thicker nanofibers (Figure 7A). After fracture, the pull-out of the layered structure was identified. The layer pull-out significantly influenced the stress and strain at break, as shown in Figure 6. The result was completely different in the TSF nanofiber mats composed of thin nanofibers (Figures 7B,C) (Benítez et al., 2013). The pull-out of the nanofiber instead of the layer appeared, thus significantly improving the breaking stress and strain. Taken together, the observed deformation behavior of TSF nanofiber mats included layer pull-out, nanofiber pull-out, and nanofiber fracture, which gave rise to the robust performance of TSF nanofiber mats.

### Contact Angle of Tussah Silk Nanofiber Mats

**FIGURE 8:**
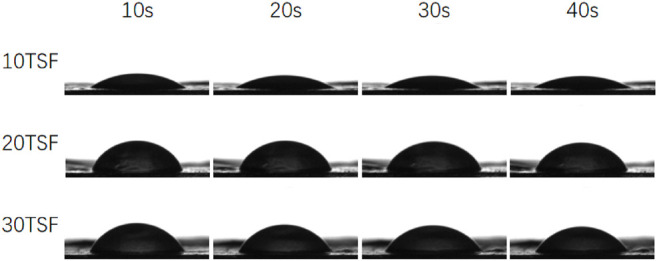
Dynamic contact angle of TSF nanofiber mats after 10-, 20-, and 30-min physical shearing.

The measurement results of contact angle are shown in Figure 8 and Table 1. It is known that the smaller contact angle represents higher hydrophilicity. The contact angles of the three kinds of mats were all within 90, which were close to that of the electrospun TSF nanofiber mats (Shao et al., 2017; Wang et al., 2017), indicating the excellent hydrophilicity of TSF nanofiber mats. The finer nanofiber had a larger specific surface area and more contact area with water on the mat surface, which endowed the nanofiber mat with more excellent hydrophilicity. However, with the increase of shearing time, the contact angle of the TSF nanofiber mats gradually increased. This was because of the decreased nanofiber diameter, which resulted in smoother surface and compact fiber structure that prevented water entering into the mats. The hydrophilic material can promote the adhesion and proliferation of cells on the surface through the absorption of nutrients (He et al., 2010; Bhattacharjee et al., 2015). Therefore, the superior hydrophilicity made the TSF nanofiber mats an excellent biomaterial.

**TABLE 1:** Dynamic contact angle of TSF nanofiber mats after 10-, 20-, and 30-min physical shearing at different times.

Sample	Contact angle at 10s (°)	Contact angle at 20s (°)	Contact angle at 30s (°)	Contact angle at 40s (°)
10TSF	40.50 ± 1.66	35.50 ± 1.50	35.25 ± 1.09	35.00 ± 0.71
20TSF	60.50 ± 4.15	60.25 ± 4.76	59.00 ± 3.08	58.25 ± 4.32
30TSF	72.86 ± 2.56	66.75 ± 0.83	57.50 ± 4.27	55.00 ± 2.24

### *In vitro* Degradability of TSF Nanofiber Mat

**FIGURE 9:**
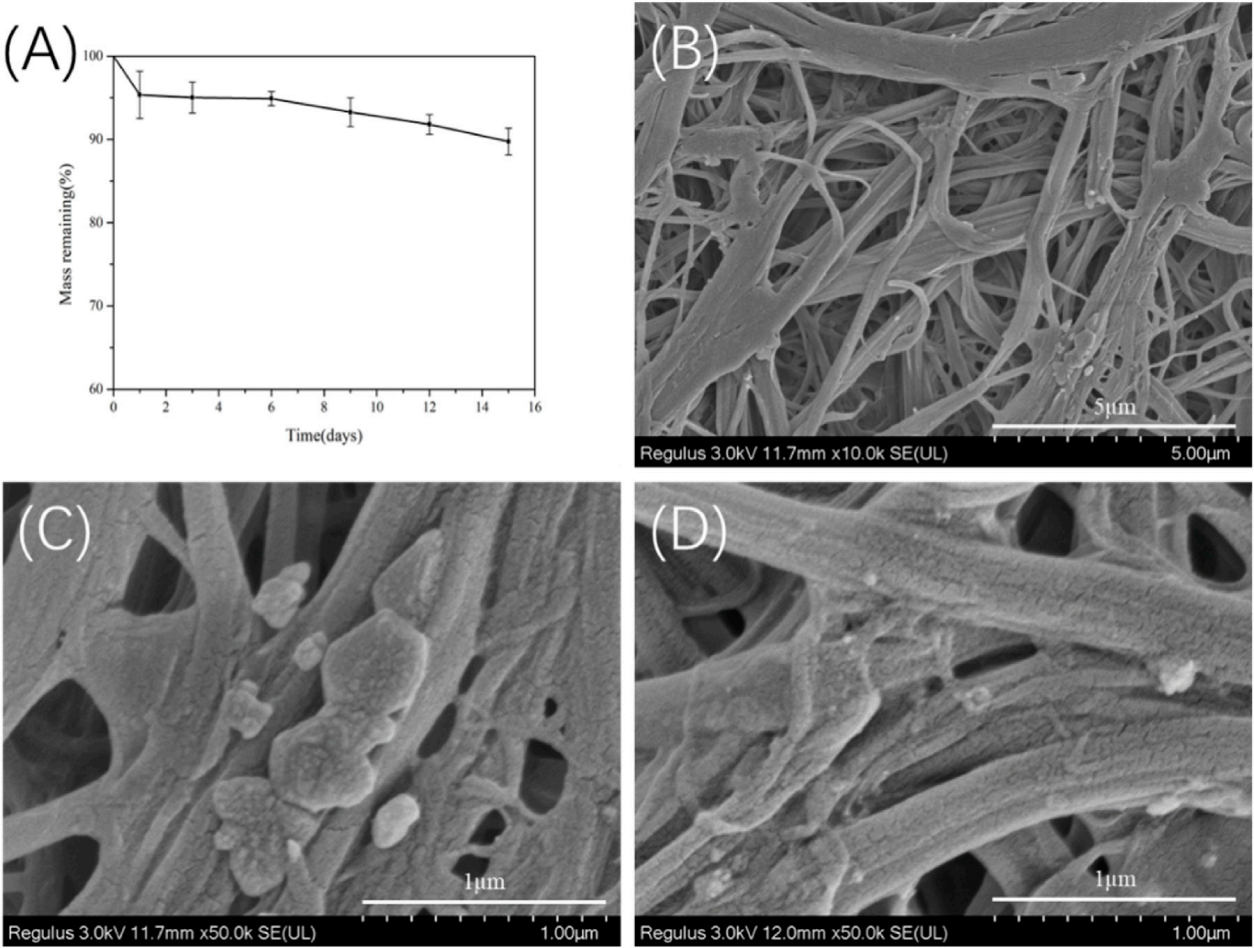
*In vitro* enzymatic degradation of the 30TSF nanofiber mat **(A)** Mass remaining versus degradation time (n = 3 per time point) **(B–D)** SEM observation of the 30TSF nanofiber mat after degradation for 15 days.

To evaluate the *in vitro* degradability, the TSF nanofiber mat derived from 30-min physical shearing was immersed in protease XIV, which is known to effectively degrade silk fibroin (Horan et al., 2005; Taddei et al., 2006). As shown in Figure 9A, the TSF nanofiber mat lost about 11% of its original weight after 15 days. SEM imaging showed that transverse and longitudinal cracking and nanoscale fragments degraded from the TSF nanofiber mat. Similar results of degraded silk fragments were reported in previous studies (Numata et al., 2010; Zhou et al., 2010; Umuhoza et al., 2020). Compared with B. mori silk, tussah silk showed enhanced stability with slow degradation due to its high crystal content (Zhao et al., 2011; Sahu et al., 2015). Therefore, the TSF nanofiber mat featured slow degradation behavior, which could provide durable support or protect cell adhesion or tissue growth (Perrone et al., 2014).

### Biocompatibility of Nanofiber Film of Tussah Silk

**FIGURE 10:**
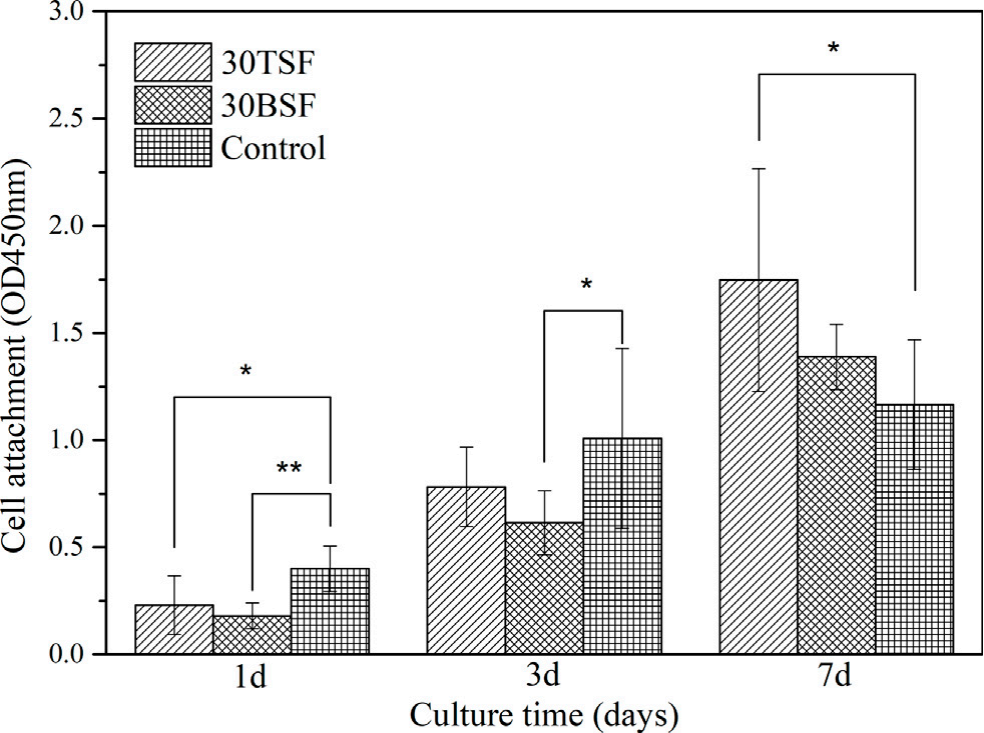
CCK-8 test for the proliferation activity of NIH-3T3 on 30TSF, 30BSF, and control (*p < 0.05, **p < 0.01).

The proliferation activity of NIH-3T3 cells on the BSF and TSF nanofiber mats was quantitatively analyzed by the CCK-8. The cultured plate was used as a control. It could be seen from Figure 10 that the cell number increased on both BSF and TSF nanofiber mats with the increase of culture time, suggesting their good biocompatibility. More cell adhesion on 1 day and more proliferated cell number on 3 and 7 day were observed on TSF nanofiber mats than those on BSF nanofiber mats. Previous studies had reported that the biocompatibility of TSF was superior to BSF, such as enhanced cell proliferation and tissue regeneration (Zhang et al., 2020). The inherent RGD sequence in TSF material was a well-known cell-adhesion site and contributed significantly to the superior biocompatibility of TSF material (Wang et al., 2019).

**FIGURE 11:**
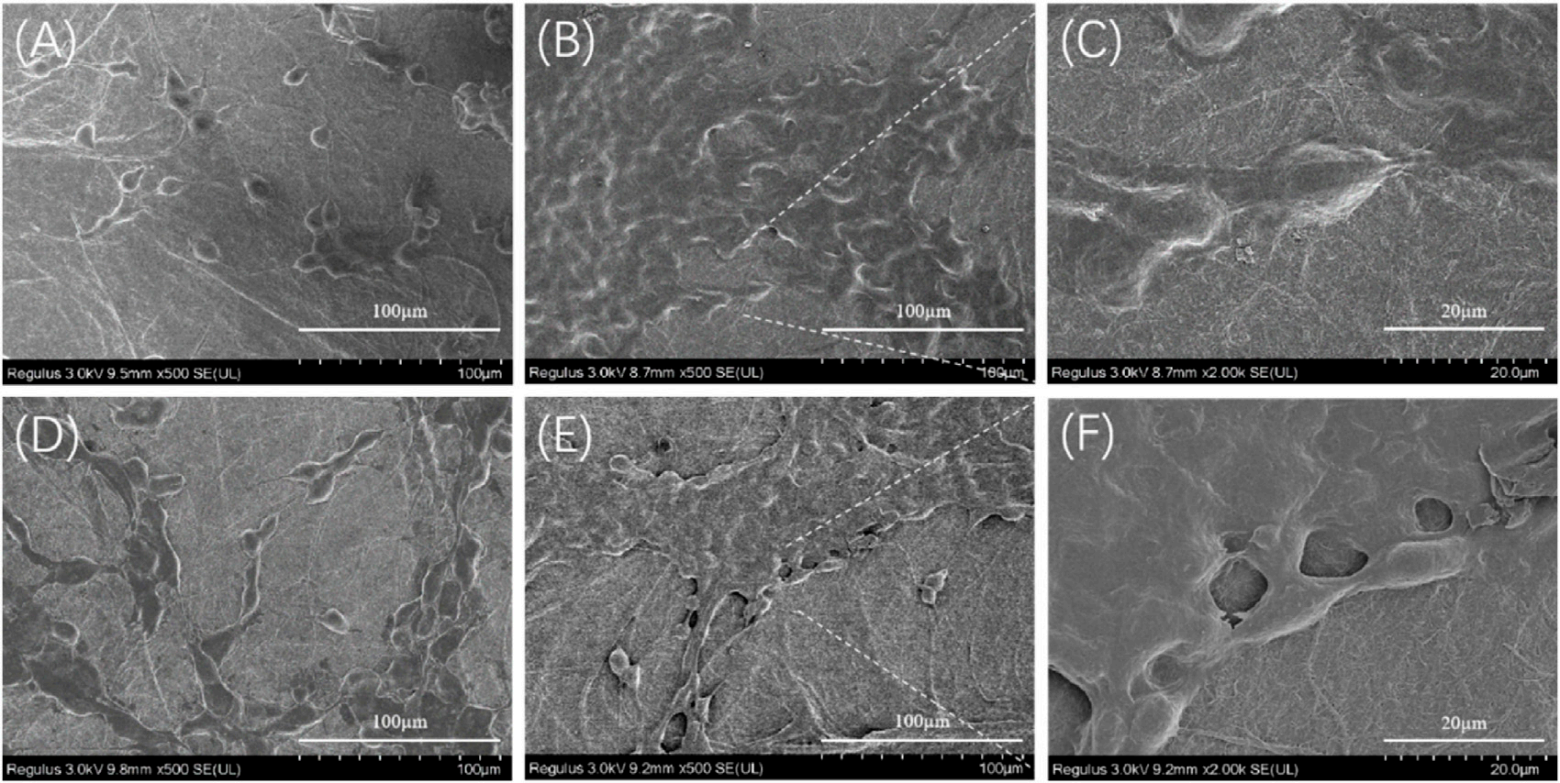
SEM images of NIH-3T3 cells growing on 30TSF and 30BSF for 3 days and 7 days **(A)** NIH-3T3 cells grown on 30TSF for 3 days **(B–C)** NIH-3T3 cells grown on 30TSF for 7 days **(D)** NIH-3T3 cells grown on 30BSF for 3 days **(E–F)** NIH-3T3 cells grown on 30BSF for 7 days.

The morphology of NIH-3T3 cells grown on nanofiber mats was observed using a scanning electron microscope, as shown in Figure 11. The BSF and TSF nanofiber mats all support cell adhesion and proliferation. After the 3-day culture, more NIH-3T3 cells and their clusters were observed on TSF nanofiber mats than those on BSF nanofiber mats. With the increase of culture time to 7 days, the number of cells on the surface of the nanofiber mats significantly increased, and the cell sheet formed on both nanofiber mats. The SEM results further confirmed the good biocompatibility of TSF nanofiber mats, which would be a promising biomaterial.

## Conclusion

The current study presents a facile method for constructing TSF nonwoven mats with nanofibrous structure, outstanding mechanical properties, biodegradability, and enhanced biocompatibility. The nanofiber with a diameter ranged from 196.5 to 139.7 nm was prepared by disintegrating TSF with physical shearing. Excellent mechanical properties were achieved for TSF nanofiber mats. The TSF nanofiber mat featured high breaking stress of 72.68 MPa because of the retention of native nanofibril structure and its compact stacking and high β-sheet crystal structure. The TSF nanofiber was biodegradable and lost more than 10% of its original weight after 15 days degradation in protease XIV. The cell experiment demonstrated that the TSF nanofibers have superior biocompatibility to BSF nanofiber mats, showing great potential application in biomedicine.

## Data Availability

The original contributions presented in the study are included in the article/Supplementary Material; further inquiries can be directed to the corresponding authors.
